# “If It’s Not Working, Why Would They Be Testing It?”: mental models of HIV vaccine trials and preventive misconception among men who have sex with men in India

**DOI:** 10.1186/1471-2458-13-731

**Published:** 2013-08-07

**Authors:** Venkatesan Chakrapani, Peter A Newman, Neeti Singhal, Ruban Nelson, Murali Shunmugam

**Affiliations:** 1 Centre for Sexuality and Health Research and Policy (C-SHaRP), 38 (Old No. 167), Ground Floor, Rangarajapuram Main Road, Kodambakkam, Chennai 600024, India; 2 Factor-Inwentash Faculty of Social Work, University of Toronto, 246 Bloor Street West, Toronto, Ontario M5S 1V4 Canada; 3 The Humsafar Trust, III floor, Manthan Plaza, Nehru Road, Vakola, Santacruz (East), Mumbai 400 055 India

**Keywords:** HIV vaccines, Clinical trials, Informed consent, Preventive misconception, Vaccine-induced seropositivity, Men who have sex with men, India, Qualitative research

## Abstract

**Background:**

Informed consent based on comprehension of potential risks and benefits is fundamental to the ethical conduct of clinical research. We explored mental models of candidate HIV vaccines and clinical trials that may impact on the feasibility and ethics of biomedical HIV prevention trials among men who have sex with men (MSM) in India.

**Methods:**

A community-based research project was designed and implemented in partnership with community-based organizations serving MSM in Chennai and Mumbai. We conducted 12 focus groups (n = 68) with diverse MSM and 14 key informant interviews with MSM community leaders/service providers using a semi-structured interview guide to explore knowledge and beliefs about HIV vaccines and clinical trials. Focus groups (60–90 minutes) and interviews (45–60 minutes) were conducted in participants’ native language (Tamil in Chennai; Marathi or Hindi in Mumbai), audio-taped, transcribed and translated into English. We explored focus group and interview data using thematic analysis and a constant comparative method, with a focus on mental models of HIV vaccines and clinical trials.

**Results:**

A mental model of HIV vaccine-induced seropositivity as “having HIV” resulted in fears of vaccine-induced infection and HIV stigma. Some participants feared inactivated vaccines might “drink blood” and “come alive”. Pervasive preventive misconception was based on a mental model of prevention trials as interventions, overestimation of likely efficacy of candidate vaccines and likelihood of being assigned to the experimental group, with expectations of protective benefits and decreased condom use. Widespread misunderstanding and lack of acceptance of placebo and random assignment supported perceptions of clinical trials as “cheating”. Key informants expressed concerns that volunteers from vulnerable Indian communities were being used as “experimental rats” to benefit high-income countries.

**Conclusions:**

Evidence-informed interventions that engage with shared mental models among potential trial volunteers, along with policies and funding mechanisms that ensure local access to products that demonstrate efficacy in trials, may support the safe and ethical implementation of HIV vaccine trials in India.

## Background

It is an ethical imperative that prospective research volunteers are to be provided adequate information about clinical trials and their potential risks and benefits to enable meaningful informed consent [1,2]. Providing information alone, however, even lay descriptions of scientific concepts, may be insufficient to support understanding and fully informed decisions about clinical trial participation [2-4]. Challenges for achieving participation based on meaningful informed consent may be even greater in clinical trials sponsored by rich countries of the Global North conducted among marginalized communities in the Global South; yet this is a necessary reality if we are to develop HIV vaccines with demonstrated efficacy in the developing world, where they are most sorely needed [5-7].

India, with a population of 1.2 billion and the second highest number of persons living with HIV in the world [8], as well as expertise in vaccine production [9], is a strategically important site for HIV vaccine research. Nevertheless, a history of some unethical clinical trials [9-11], challenges surrounding the implementation of Phase I HIV vaccine trials [12-14] and ongoing controversy over roll-out of licensed vaccines in India [15-22] suggest the critical importance of formative research to support the ethical conduct of HIV vaccine trials based on meaningful informed consent. In particular, “social resistance and rumours” have been documented in regard to oral polio vaccines, founded on mistrust of vaccine efficacy and the government’s intentions [15,16]. Controversies have also erupted in response to a demonstration project that administered human papillomavirus vaccine to 23,000 adolescent girls in two Indian states, documented in both medical journals [17,18] and the media [20-22]. The deaths of four girls were alleged to be vaccine-related, with further contentions that participants did not fully comprehend the risks of vaccination despite signing a consent form.

Further challenges may arise in conducting HIV vaccine trials with marginalised communities. In India, HIV prevalence among men who have sex with men (MSM) (7.4%) is more than 20-fold higher than the general population (0.3%) [8]. A concentrated epidemic among MSM makes this a potentially viable community for HIV vaccine trials. However given that adult consensual same-sex sexual behaviour was a criminal offence in India until 2009 [23], and stigma and discrimination against MSM is still rampant in general society and healthcare settings [24], participating in HIV prevention trials may engender particular concerns among MSM [3].

### Mental models

Comprehension of new information is contingent on how that information fits within pre-existing mental models [25-27]. Mental models are simplified cognitive representations of complex external realities; they may be incomplete, but are useful in guiding reasoning, decision-making and behaviour [25,26,28]. A mental models approach has been applied in health and environmental risk domains to support informed decision-making and promote safer behaviours [26,28]. Mental models have also been elicited to understand fears and misconceptions regarding licensed vaccines and thereby to support strategies to increase uptake [29,30]. For example, a mental models approach indicated that even parents who reported generally positive attitudes towards childhood vaccination expressed lingering concerns and misconceptions about the need for multiple injections, which were infused with misinformation spread by anti-vaccine propaganda [30]. The identification of particular knowledge gaps supported the need to provide clear lay language explanations that integrated with parents’ mental models in order to counter misinformation about vaccination risks.

A mental models approach, however, is new to the context of clinical trials. Mental models regarding experimental vaccines in clinical trials may share similarities with those of licensed vaccines, for example, in regard to the need for multiple injections; however, participating in placebo-controlled trials is also quite distinct from uptake of vaccines that have already been approved for public licensure [27,31]. Identifying and understanding mental models --individuals’ existing web of knowledge regarding HIV vaccines and clinical trials-- may support the development of effective communication strategies to facilitate community engagement and meaningful informed consent, as well as recruitment and retention, in ethically conducted clinical trials. With Phase II HIV vaccine trials planned among MSM in India [9], we explored mental models of candidate HIV vaccines and clinical trials.

## Methods

The study protocol was approved by the Institutional Review Boards of the University of Toronto and The Humsafar Trust, Mumbai. All participants provided written informed consent.

We used a community-based research approach [32]; community-based organizations (CBOs) serving MSM in Chennai (Social Welfare Association for Men and Sahodaran) and Mumbai (The Humsafar Trust) collaborated throughout all stages of the research. We conducted focus groups with MSM service users and MSM peer outreach educators from local CBOs in each city. Trained peer outreach staff recruited focus group participants by word of mouth, mitigating potential risks of printed materials indicating MSM and HIV. We conducted key informant (KI) interviews with MSM community leaders, CBO staff and healthcare providers in each city.

Eligibility criteria for focus group participants were self-identification as MSM, aged 18 years or above and ability to provide informed consent. We used stratified purposive sampling to include perspectives of different MSM subgroups, with separate focus groups for kothis (feminine gender expression and generally receptive partners in anal sex), panthis (masculine gender expression and generally insertive partners in anal sex), and double-deckers (both insertive and receptive roles). In Chennai, CBO peer outreach educators had previously taken part in community consultation meetings sponsored by the International AIDS Vaccine Initiative (IAVI), which was exploring the feasibility of conducting future HIV vaccine trials in India. We used purposive sampling to select information-rich [33] key informants with in depth knowledge of the study populations.

### Data collection

We used a semi-structured interview guide to explore knowledge and beliefs about HIV vaccines and clinical trials. Focus groups (60–90 minutes) and interviews (45–60 minutes) were conducted in participants’ native language (Tamil in Chennai; Marathi or Hindi in Mumbai); a few KIs chose to be interviewed in English. Focus groups and interviews were audio-taped, transcribed verbatim, redacted and translated into English. We checked the accuracy of transcripts by randomly selecting 20% and comparing them with the respective audio files.

### Data analysis

Focus group and interview data were explored using thematic analysis [34] and a constant comparative method [35], with a focus on possible mental models of HIV vaccines and clinical trials. We developed a code book based on the interview guide and available literature and added codes that emerged during analysis. Two bilingual investigators individually analysed each transcript, followed by team analysis using NVivo 7. Differences in coding were resolved by consensus in research team meetings. We sought to identify pre-existing mental models and traced how they facilitated or hindered comprehension of basic scientific information regarding HIV vaccine trials. Member checking was conducted by discussing findings and interpretations in meetings with field research teams and community leaders in each site [36], with attention to differences in perspectives and interpretations between key informant community leaders/service providers and MSM CBO clients.

## Results

### Sociodemographic characteristics

We conducted seven focus groups (n = 43) in Chennai and five (n = 25) in Mumbai. Participants’ mean age was 28 years. The majority (52%) of focus group participants identified as kothi, one-fifth (21%) as double-deckers and about one-fifth (18%) panthis; 16% (n = 11) were married. Notably, due to powerful sociocultural and familial pressures in India, a substantial proportion of self-identified MSM are married to women [3]. Most participants were of lower socioeconomic status. Key informants’ (n = 14) (mean age = 40 years) were MSM CBO staff (n = 8) and leaders (n = 4), and healthcare providers (n = 2) working with MSM, all with college degrees. Additional sociodemographic characteristics are reported in Table 1.

**Table 1:** Sociodemographic characteristics of focus group participants in Chennai and Mumbai, India (n = 68)

**Characteristic**		**n**	**%**
Age (years)	Mean	28	--
	Range	20-46	--
Marital status	Unmarried	57	84
	Married	11	16
Education	Primary (<= 5th grade)	10	15
	6th - 11th grade	22	32
	High school degree	18	26
	College degree	18	26
Employment	Community agency staff	15	22
	Daily wage labourer	16	24
	Private company staff	19	28
	Sex work	8	12
	Unemployed	10	15
Sexual identity	Kothi	35	52
	Double-decker	14	21
	Panthi	12	18
	Versatile/Bisexual	4	6
	Other	3	4

### Vaccine-induced seropositivity (VISP) and fear of vaccine-induced infection

**Figure 1:**
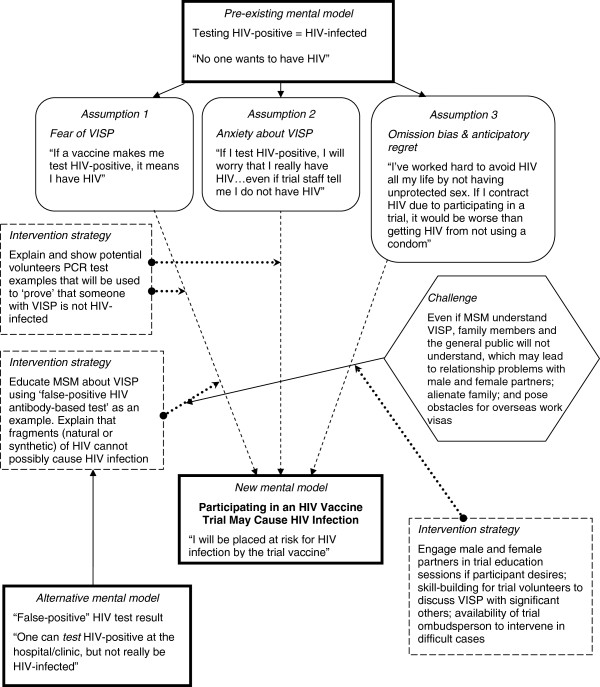
Mental model of HIV vaccine-induced seropositivity among MSM in Chennai and Mumbai, India (n = 82).

A pervasive mental model was that testing HIV positive as a response to a candidate vaccine signified actual infection. Figure 1 depicts a mental model of VISP and potential challenges as well as strategies to improve comprehension. Participants were largely unaware of VISP, except for two peer educators who had been involved as peer research interviewers in a formative research study related to HIV vaccines. Testing “positive for HIV” was equated with being infected (“there is HIV”), as participants were familiar with HIV antibody testing offered in government voluntary HIV testing centres. The first time the concept of VISP was explained by the facilitator, participants did not comprehend it as *testing* positive for HIV with no actual HIV infection. After additional explanation, even those who eventually understood VISP had concerns about how others would react to their testing HIV-positive. Participants described situations in which trial volunteers might be screened for HIV outside of a trial: prevention-of-parent-to-child-transmission (PPTCT) programs in which the husband is encouraged to be tested for HIV along with his wife; and pre-employment HIV screening when applying for jobs abroad.

There are chances for MSM to go to PPTCT centre during wife’s pregnancy; there are chances for both of them to undergo HIV testing. At that time if he is found to be ‘HIV positive’, what would happen? (Kothi, FG2, Chennai)

Focus group facilitators gave brief explanations of candidate vaccines that may contain viral fragments or possibly inactivated (killed) whole viruses. One participant questioned: “You said dead virus is put in. How do we know? … After going in it drinks blood and becomes alive, then?” (Kothi, FG6, Chennai).

The possibility of being infected by a candidate vaccine led to trepidation among participants: “I am HIV-negative and I have kept myself free of HIV for all these years…If I become HIV-positive because of a [test] vaccine I’ll be punished for a lifetime. I am scared about that” (Kothi, FG1, Mumbai). Even with understanding of VISP, a participant explained the tremendous fear associated with HIV: “One will definitely be shocked even if his result is false-positive. Yes, a good and strong person may not be afraid of anything but HIV. They are afraid of only one word – ‘HIV’. Do you get it?” (Double-decker, FG3, Chennai).

A kothi-identified peer educator similarly reported: “We have explained about this [VISP], but whether after knowing about VISP they [MSM community] would participate or not, we don’t know; because everyone is afraid of testing positive for HIV” (KI4, Chennai).

A participant expressed hope that “we can explain this [VISP] to people,” as some MSM are already familiar with the idea of “false-positive HIV tests”: “We can tell them that similar to the possibilities of having a false-positive HIV test result, even if one’s result comes as HIV-positive, he is not really infected. We know they won’t lament then, okay?” (Double-decker, FG3, Chennai).

Another participant suggested using the media to challenge mental models, and provided an example from a well-known media campaign– “pulli rajavukku AIDS varumaa” –roughly translated as, “Will a lucky man [who is popular among women] get AIDS?” This campaign in Tamil Nadu challenged the popular notion that some persons will not get HIV even if they engage in risky behaviours, because they are lucky.

If it is explained people will definitely understand. Because people did not know much about HIV 7 or 10 years back, the advertisement “pulli rajavukku AIDS varumaa” was frequently broadcasted in the TV for a month’s period when people could not immediately understand its essence. Later on many got to know about the concept since it was broadcasted in all TV channels. So, similarly if you explain about the vaccine trials they will come to know about it. So, the message needs to tell the people that there are chances for false-positive because of participating in such research. This would really help them to believe that they are negative even if they are tested positive. (FG5, Chennai)

### Preventive misconception and behavioural disinhibition

**Figure 2:**
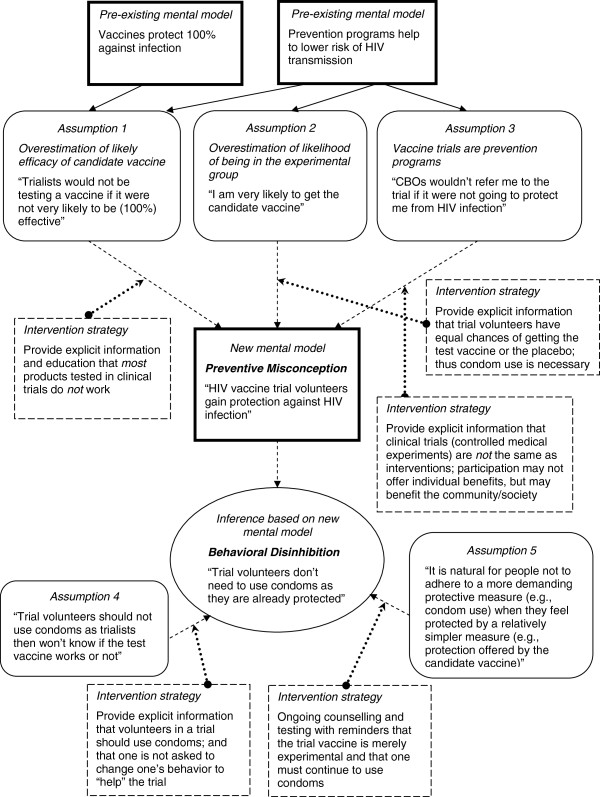
Mental model of preventive misconception and behavioural disinhibition among MSM in Chennai and Mumbai, India (n = 82).

A common mental model was that participation in biomedical HIV prevention trials provides protection against HIV infection. Figure 2 illustrates a mental model of preventive misconception, the belief that one gains protection by virtue of being enrolled in a prevention trial [37]. This widespread belief was founded on three fundamental misunderstandings: first, overestimation of the likely effectiveness of a candidate vaccine; second, lack of comprehension of “placebo” and randomization, and overestimation of one’s chances of being assigned to the experimental arm; and; third, that prevention trials are intervention programs.

Participants specifically indicated belief in the efficacy of candidate vaccines tested in clinical trials. A peer educator explained that trial participants would think, “If it [candidate vaccine] is not working, then why would they [trialists] be actually testing it?” (KI6, Chennai).

Peer outreach workers and a program director of an HIV intervention project for MSM joked that once an HIV vaccine is available for public use they will be unemployed. Although intended in jest, this provides further evidence of a mental model of HIV vaccines as nearly 100% effective; therefore there will be no further need for condom education or distribution:

If there is no vaccine then there is no choice other than condom; condom is a must. If vaccine is available, condom use will become unimportant. One will think “I have been vaccinated so I would not require a condom.” (FG1, Chennai)

Another key informant provided a possible explanation for belief in the efficacy of candidate vaccines in clinical trials: “Usually one tends to believe that ‘something is better than nothing’. So, even if the volunteers were informed that [trial] vaccine may or may not protect one from getting HIV, they would at least expect some protection from it.” (KI2, Chennai).

Participants also explained that people will opt for easier and less taxing ways to prevent HIV; accordingly, decreases in condom use among trial volunteers were seen as “natural”, “normal”, “to be expected”, and even quite logical: “Now that one is on vaccine, condoms will be seen as an additional burden. People usually think like that, right?” (Kothi, FG2, Chennai).

Participants described the likelihood of widespread behavioural disinhibition due to preventive misconception among trial volunteers resulting in less consistent condom use for anal sex:

Kothis will not be careful in using condoms with panthis. Once they are injected with vaccine, kothis will become fully confident that they would never get infection. So they will not insist their panthis to use condoms or they would avoid using condoms. (Kothi, FG2, Chennai)

When I get the vaccine I will feel that nothing is going to happen to me. I can have sex with whomever I want. (Kothi, FG1, Mumbai)

Kothis [in sex work] would think “I won’t get HIV because I have been given vaccine”. So they will not use condoms in their sex work. (KI3, Chennai)

The possibility that condom use among trial volunteers may decrease posed a dilemma for community leaders who manage HIV intervention programs. A community leader in Chennai explained:

Yes, we want our community to be free of HIV. To develop a vaccine one needs people to participate. But what if then people do not want to use condoms? The work we have put in for several years to promote condom use among MSM will then be wasted. (KI1, Chennai)

### Placebo-controlled trials and random assignment

None of the focus group participants had heard of “placebo” before. Facilitators had difficulty in explaining what a placebo is and why it is needed; the concept was considered too technical to be explained in simple terms and it was difficult to find suitable words or examples in the vernacular of participants. Even after the facilitator explained why a placebo and control group were needed to conduct a clinical trial, participants reported that most trial volunteers would not consistently use condoms due to overestimating their likelihood of being in the experimental vaccine group.

Participants reported the perception that double-blind placebo-controlled trials amount to “cheating”. They further reasoned that unblinding volunteers who received placebo would make these volunteers less likely to become careless and engage in sexual risk behaviours. Therefore, it was seen as unethical on the part of trialists not to disclose group assignment. This assertion suggests that preventive misconception is to be expected among trial volunteers, and further presumes that most volunteers would overestimate experimental vaccine efficacy.

Peer educators and community leaders reported that if even they, in spite of their long-term experience “in the field of HIV”, found it difficult to understand the concept of placebo, then MSM from the “grassroots level” would definitely have difficulty in understanding it. They reported that explaining double-blind assignment to volunteers would pose problems for trial recruitment and retention:

Community will not accept if we tell like this; even people who accepted [to participate] earlier will now not accept, because they would be afraid that they are being cheated…they would not understand. (Kothi, FG6, Chennai)

[Trial participants] may not know much information about placebo…They will believe that “I have been given HIV vaccine, I can do whatever I want…” (KI9, Chennai)

A key informant further indicated not understanding how one can evaluate vaccine efficacy if everyone is counselled to use condoms:

If you are not going to tell who is on placebo and who is on HIV vaccine, then how would you know whether vaccine is working or not as everyone is counselled to use condoms? (KI1, Chennai)

Similarly, a key informant indicated suspicions that the reason for blinding and a placebo was to deliberately mislead volunteers that they are in the experimental vaccine group so that they will not use condoms consistently, which will then help in assessing vaccine efficacy.

### Disclosure of STEP study outcomes to prospective trial volunteers

Toward the end of the interview, facilitators and interviewers provided brief information about the STEP study in order to explore participants’ perspectives on what information should be shared with future trial volunteers. Although this Phase IIb HIV vaccine trial with people at high risk of HIV infection did not include India, the international multi-site trial was terminated early when it was determined that the experimental vaccine would not prove to be efficacious. Subsequently, it was shown that a subgroup of trial volunteers who had received the experimental vaccine were placed at higher risk for HIV infection than the placebo group [38,39]. In response to the STEP study, broad changes have been implemented to better ensure the safety of trial participants and experimental vaccines to be tested; nevertheless, the unexpected harms to volunteers in a clinical trial raised significant and widely discussed challenges for HIV vaccine testing and development.

Some MSM reported that trialists should disclose everything about the STEP study so that MSM can decide for themselves whether or not to participate. Not disclosing STEP trial outcomes amounted to “cheating”:

We have to share it openly and only those who still want to participate should then be referred; otherwise it is wrong. (Kothi, FG6, Chennai)

They should not lie because they think there would be *Beeli* [problem] if they tell the details. Then it is like misusing us; all information should be definitely revealed. (KI1, Chennai)

This information you should give so that later they don’t blame [trialists] by saying, “Why didn’t you tell us first?” (Panthi, FG5, Mumbai)

Alternately, some participants and community leaders thought that because the STEP trial happened in other countries and the same candidate vaccine is not going to be tested in India (information provided by the facilitator/interviewer), one need not disclose anything about STEP. They further indicated that if this information were provided then MSM would become afraid and not want to participate at all: “People will be confused and will not understand; so don’t tell”. (KI2, Chennai).

After hearing about the outcomes of the STEP study, a key informant asserted that vaccine trials are conducted in India because trialists see Indians as “guinea pigs”. He described this as ‘neocolonialism,’ presuming that rich countries largely benefit from the results of these trials:

Most of these trials are deliberately conducted among people who are economically disadvantaged and who are from developing countries…I even doubt whether Phase I trial [in India] among normal human volunteers was actually conducted. (KI9, Chennai)

Doubt regarding the past implementation of a Phase I trial (among healthy volunteers) in fact conducted in India [9] was founded on the belief that no one from the middle or upper classes would have volunteered for an HIV vaccine trial.

A community leader similarly asked, “Whether trials are happening in other countries?…Why this need to be conducted among MSM in India, and not among FSWs [female sex workers] or IDUs [injecting drug users]?” He later expanded on this, saying that he posed these questions to the interviewer because he needs to respond to the MSM communities he serves if they raise these questions. If not, he added, clients of his agency would think he had “sold” them to trial sponsors.

In contrast to key informants, focus group participants rarely questioned the motivations behind the HIV vaccine trials planned with Indian MSM. One participant indicated he was against such trials as he felt they would only increase the negative image that the general public has of MSM as a ‘high risk group’, and he also did not want MSM to be treated as “experimental rats”.

## Discussion

This study among Indian MSM revealed several common mental models regarding HIV vaccine trials, some of which indicated misunderstanding and misconceptions of procedures fundamental to clinical research. With Phase II trials planned among MSM in India in the near future, understanding potential volunteers’ perspectives on candidate HIV vaccines and vaccine trials may yield insights to support meaningful informed consent, recruitment and retention, and the safe and ethical conduct of these trials.

A major concern emerged in relation to VISP, which was perceived as a deterrent to otherwise willing MSM volunteers participating in trials. The mental model that an HIV-positive test result is the same as HIV-infection, founded on familiarity with commonly used antibody-based HIV tests in India, posed difficulties in understanding that one can be HIV-positive but not HIV-infected. Vaccine-induced infection, in addition to being feared in its own right, also resulted in the anticipation of greater regret than if one had contracted HIV due to unprotected sex. This line of thinking is consistent with omission bias, a frequently observed cognitive bias in decision-making in which harms (i.e., HIV infection) due to acts of commission (i.e., trial participation) tend to result in greater regret than harms due to acts of omission (i.e., not using a condom) [40,41].

Secondly, participants indicated concern about situations requiring HIV testing in that others (e.g., wives, foreign employers, healthcare providers) may not accept that they are not actually HIV infected. VISP was similarly a primary barrier to HIV vaccine trial participation among MSM who screened in as eligible but declined to participate in a Phase IIb trial in North America; however, the main concerns were about social consequences of VISP rather than conflation of VISP with actual infection [42]. Concerns about VISP may be exacerbated among MSM in India due to pervasive sexual stigma and related presumptions that all MSM are living with HIV (e.g., symbolic stigma) [43].

Participants also suggested strategies to impart understanding of VISP based on an alternate mental model derived from false-positive HIV tests, a concept with which many MSM are familiar (see Figure 1). This suggests that the pre-existing web of knowledge that may fuel misunderstanding may be marshalled to correct misperceptions about a particular concept (such as VISP), accurate understanding of which in turn may influence trial participation. Importantly, this also suggests that possible strategies to engage with mental models in order to correct misperceptions may emerge from communities themselves.

Although fear of vaccine-induced infection has been documented in studies of individuals who screened in but declined to participate in an HIV vaccine trial [27] and most-at-risk populations for HIV infection [44], it did not emerge as a major issue in focus group discussions. A few participants indicated apprehensions that live virus might emerge from inactivated virus vaccines and infect people. Rather, participants’ concerns were primarily focused on how to detect actual infection in a trial volunteer whose HIV-positive antibody test result might be mistaken as VISP. These concerns may be addressed by trialists through educational interventions for potential trial volunteers and by ensuring free access to viral nucleic acid-based diagnostic tests, such as polymerase chain reaction (PCR) tests [42,45].

Of particular concern, preventive misconception was widely in evidence among study participants, supported by a mental model of prevention trials as synonymous with preventive interventions (Figure 2). Some participants reasoned that MSM advocates from CBOs wouldn’t refer them to a trial if there weren’t some personal benefit for participants. Participants reported that as a result, MSM trial volunteers would exhibit behavioural disinhibition through less consistent use of condoms. We identified overestimation of the likelihood of a candidate vaccine being effective in offering personal protection—a belief in the efficacy of candidate vaccines—based on a mental model of vaccines as 100% effective as well as overestimation of assignment to the experimental group. Several different rationales were revealed in support of these beliefs: 1) Vaccines, in general, were believed to provide complete protection against infection; therefore, candidate HIV vaccines were seen as likely to protect trial volunteers from HIV infection; 2) Participants reasoned that trialists would not be spending time and resources on testing a product that is not very likely to be effective; 3) Some participants articulated that trusted CBOs, with longstanding records of providing referrals to competent health and social services, would not refer clients to participate in clinical trials if the products to be tested were not going to be effective; and, 4) The view that “something is better than nothing”; so by merely participating in the trial, one is necessarily safer than in not participating.

Participants also demonstrated widespread lack of understanding of “placebo” as well as the rationale for a control group; once these concepts were explained by the interviewer, several participants and key informants still indicated overestimation of the likelihood of being assigned to the experimental group, which supported preventive misconception. Thus, mental models of prevention trials as interventions and vaccines as completely efficacious, along with lack of understanding of placebo and random assignment, were reported as likely to lead to widespread behavioural disinhibition among MSM trial volunteers.

Preventive misconception constitutes what may be an inappropriate motivation for participating in a clinical trial based on lack of comprehension of the trial’s purpose or a sense of invincibility [44,46,47]. Importantly, this misconception may enable behavioural disinhibition, resulting in increased risk to participants [37,47,48]. The mental model of preventive misconception (Figure 2) suggests that intervention strategies to challenge misconceptions about experimental vaccine efficacy and randomization, and thereby to mitigate the potential for increased risk behaviours (e.g., not using a condom) based on those beliefs, should include provision of explicit and consistently reinforced information and education to the effect that: 1) clinical trials are not the same as interventions; 2) most products tested in clinical trials do not work; and, 3) trial volunteers have equal chances of getting the test vaccine or the placebo.

The particular sociocultural context of kothis in India also may impact on behavioural disinhibition: the perceived opportunity to forego condom use may be experienced as consonant with kothis’ feminine gender expression and desire to attract panthis, in addition to being incentivized by saving on the cost of condoms and averting logistical challenges of negotiating condom use. Interestingly, participants invoked a former Indian AIDS education media campaign—which used a macho character to challenge the notion that one avoids HIV by being “lucky” rather than by using condoms—as a possible model for mitigating preventive misconception. The specific beliefs that contribute to preventive misconception could be integrated into such a campaign as targets for change.

Another mental model that emerged across participants was the belief that it is wrong to conceal something that might harm someone, with a strong value placed on transparency. This led to criticisms of the very basis for randomised controlled trials (RCTs), in the use of placebo-controls and double-blinding, and further influenced opinions on the importance of providing information about the outcomes of the STEP study to all potential HIV vaccine trial volunteers.

An additional permutation on the theme of transparency was invoked by key informants, who suspected that the reason for implementation of HIV vaccine trials in India is that foreign institutions want to benefit by using low socioeconomic, marginalised Indian populations as ‘guinea pigs’. In fact, conducting clinical trials in India requires technical and ethical clearance from the government, with approval by the Indian Council of Medical Research (ICMR). However, the perception among key informants, who had higher education and occupied leadership roles, that vulnerable Indian people were being used as guinea pigs may be founded on injustices and economic motivations reminiscent of the colonial era and perceived Indian governmental indifference to marginalised communities [9]. The disenfranchisement of MSM from mainstream Indian society may make issues of mistrust more salient in their mental models of HIV vaccine trials.

Mistrust regarding clinical trials raises the importance of explicitly addressing doubts in the minds of both MSM community leaders and grassroots level MSM about the motivations behind HIV vaccine trials and who is sponsoring the trial, along with demonstrating how the trial may benefit Indian MSM and other marginalised communities. Distrust of foreign sponsors, in particular, supports the value of having policies and funding mechanisms in place to enable access in low- and middle-income countries, particularly among trial volunteers, to HIV vaccines that demonstrate efficacy in clinical trials, once approved and licensed [6].

Further challenges remain in how to inculcate research literacy, including basic comprehension of concepts such as placebo, randomization and the scientific rationale for RCTs, as well as how to share information about possible risks due to unpredictable negative consequences (like those in the STEP study) without unnecessarily scaring potential participants. A strategy informed by mental models might use the analogy of side effects of licensed medications, for example, to help explain unpredictable adverse effects of experimental vaccines. In the Indian context, building on a commonly used expression in Tamil, which can be translated as “effective medicine will taste bitter”, also might help to communicate risks of potential side effects to low socioeconomic status MSM.

Key informants largely corroborated the perspectives of focus group participants drawn from the clientele of MSM CBOs, including concerns about VISP, preventive misconception and difficulty in understanding the need for random assignment and placebo. Notably, ideas for possible solutions for resolving misunderstandings (e.g., in relation to VISP) emerged from both key informants and MSM clients. Different perspectives were revealed in key informants being more likely to insist on the importance of disclosing and explaining the STEP study outcomes to future trial volunteers—a position aligned with the ethical basis of informed consent [49]—and more likely to question the motivations of outsiders (e.g., multinational corporations and foreign governments) in conducting trials among Indian MSM.

### Limitations

As a qualitative study using purposive sampling, our findings may not apply across MSM populations in India; our intention was to understand in-depth rather than to generalise. In particular, the study sample may be more representative of lower socioeconomic MSM with kothi, panthi and double-decker identities who use CBO services; middle- and upper-class MSM, particularly those who have gay or bisexual identities, and MSM who do not access CBO services, may have different mental models of concepts related to HIV vaccines and clinical trials. Further research also may help to determine potential differences in mental models of HIV vaccines and clinical trials across different subgroups of MSM. An additional limitation is that participant responses may differ in response to an actual HIV vaccine trial; however our purpose was to anticipate challenges to comprehension and potential risks in advance of trial implementation.

### Implications for practice and research

The evidence of broadly shared mental models related to HIV vaccines and clinical trials suggests a mental models approach may be used to support and augment efforts to inculcate scientific literacy in order to promote the ethical conduct of future HIV vaccine trials in India. Mental models may be used to impart new information that is integrated with pre-existing knowledge, as in the case of explaining VISP based on comprehension of false-positive HIV tests. Accurate assessment of the bases for potential ethical challenges in clinical research is important to effectively mitigate risks and to support meaningful informed consent [46].

Nevertheless, mental models should be mobilised with reasonable caution, as indicated by a case study from South Africa: well-meaning foreign researchers may have mistakenly promoted folk beliefs among local communities about witchcraft as the source of HIV, thereby fuelling HIV stigma and even violence, in using what appeared to be an apt analogy of a snake to explain how antiretroviral medications work [50]. However, the conclusion offered in the case study that suggests one must choose either to inculcate scientific literacy or to adopt a mental models approach appears to be oversimplified. The present study suggests that efforts to promote scientific literacy alone may not be sufficient to confer understanding and acceptance of the rationale for placebos, control groups and randomization in clinical trials, nor to disavow participants and local communities of preventive misconception, or to mitigate the impact of mistrust founded on a history of colonial era abuses of medical research. A mental models approach may reveal particular misconceptions and the underlying architecture of beliefs to help guide the focus and content of scientific literacy efforts and to augment those efforts in order to improve comprehension and acceptance of the legitimacy of scientific concepts.

Importantly, the basis for preventive misconception in the present study seems to be broader than that indicated in previous empirical research conducted in the U.S. [37]. In addition to overestimating the likely personal effectiveness of an experimental vaccine and overestimating one’s chances of assignment to the experimental arm, we identified pervasive misunderstanding and lack of acceptance of placebo and random assignment, imputations that trialists and CBO referral sources alike must think the product will work as otherwise they would not be expending considerable resources and encouraging potential participants to volunteer, and a general belief that HIV prevention trials are intervention programs.

The present study suggests that engaging in discussion of the potential benefits and risks of clinical trials from the perspectives and belief systems of local communities, supporting policies and funding mechanisms that ensure access among trial volunteers to products that demonstrate efficacy in trials, as well as inculcating scientific literacy may help to advance the highest ethical standards in conducting research among MSM in India. Possible sociocultural differences in belief systems and the bases for trial misconceptions by population and geography suggest the importance of conducting formative research *in situ* before launching clinical trials, and the usefulness of a mental models approach.

Finally, although present-day trials in India are subject to government approval and ethical and safety standards, the present data suggest the importance of engaging with mental models of medical research that may be based on colonialist histories with clinical trial participants seen as “guinea pigs” or “experimental rats”, rather than merely attempting to controvert these beliefs through science. Interestingly these beliefs tended to arise more among the *most* educated among the sample, some of whom are advocates and service providers who viewed themselves as protecting marginalised communities, rather than among MSM clients themselves. Nevertheless, these key informants may be a highly important constituency to ensure effective community engagement through civil society representatives, which may be a key element in the successful implementation of biomedical HIV prevention trials [51,52].

## Conclusion

This qualitative investigation elicited mental models among MSM in India that may raise challenges for recruitment efforts and the ethical implementation of HIV vaccine trials. By understanding and mapping the existing web of knowledge in regard to HIV vaccines and clinical trials, and how it may be mobilised to support as well as challenge misinterpretations, we may develop evidence informed communication strategies to impart accurate, comprehensible and acceptable information to marginalised communities, such as MSM, in which biomedical HIV prevention trials are planned.

## Abbreviations

MSM: Men who have sex with men; CBO: Community-based organization; KI: Key informant; IAVI: International AIDS Vaccine Initiative; VISP: Vaccine-induced seropositivity; PPTCT: Prevention-of-parent-to-child-transmission; FSW: Female sex worker; IDU: Injecting drug user; PCR: Polymerase chain reaction; RCT: Randomised controlled trial; ICMR: Indian Council of Medical Research.

## Competing interests

The authors declare that they have no competing interests.

## Authors’ contributions

VC designed the study, supervised data collection and analysis, and drafted the manuscript. PAN designed the study, consulted on data collection and analysis, and wrote the final manuscript. NS implemented and supervised data collection in Mumbai and contributed to data analysis and drafting the results. RN implemented and supervised data collection in Chennai and assisted in interpretation of the results. MS coordinated data collection and contributed to data analysis and interpretation of the results. All authors read and approved the final manuscript.

## Pre-publication history

The pre-publication history for this paper can be accessed here:

http://www.biomedcentral.com/1471-2458/13/731/prepub
